# Supplementary figures and images for: The interplay of atoh1 genes in the lower rhombic lip during hindbrain morphogenesis
Source: PLoS One. 2020 Feb 3;15(2):e0228225. doi: 10.1371/journal.pone.0228225 (PMC6996848; doi:10.1371/journal.pone.0228225)

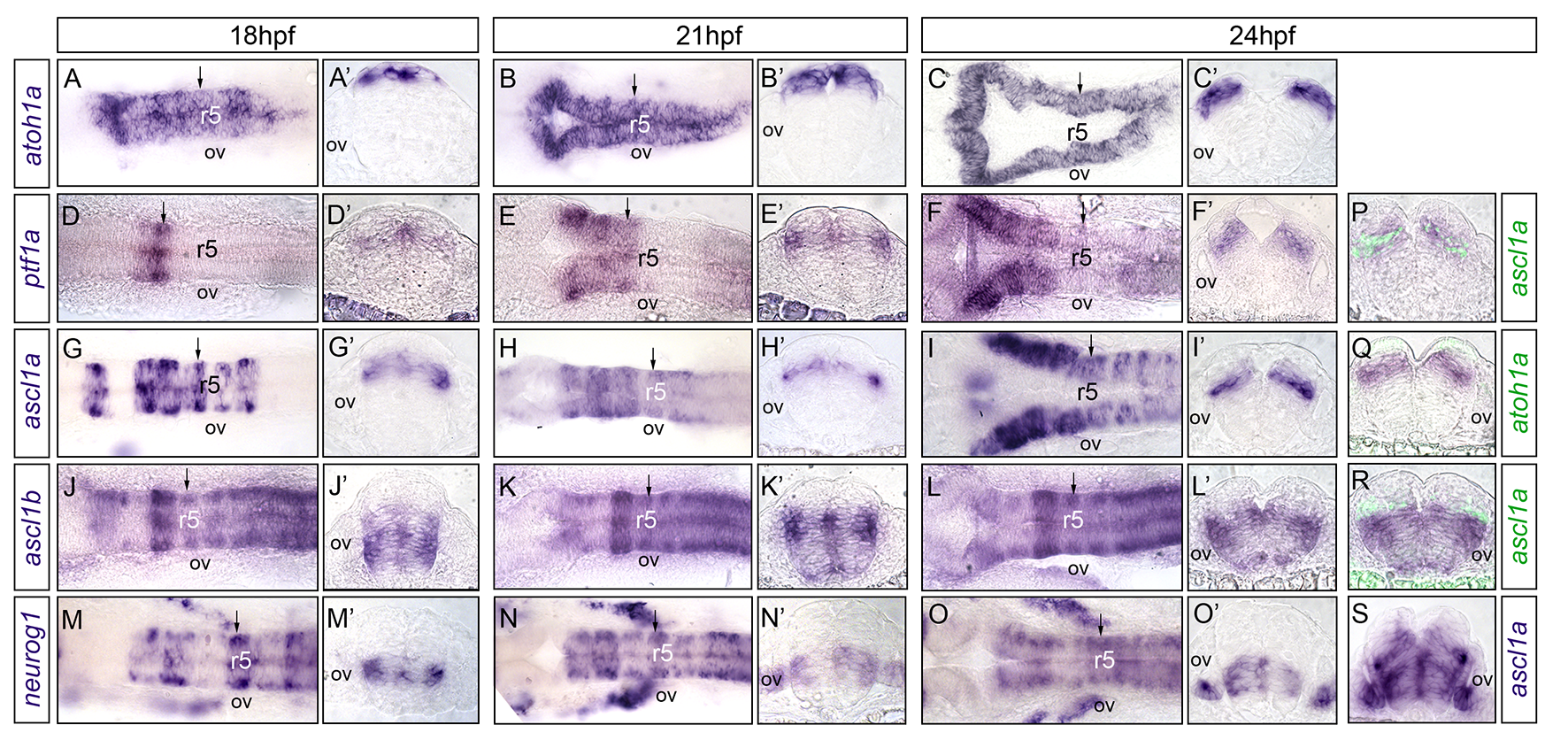

Supplement: S1 Fig — Whole mount in situ hybridization at 18hpf, 21hpf and 24hpf using atoh1a (A-C, Q), ptf1a (D-F, P), ascl1a (G-I, P-S), ascl1b (J-L, R) and neurog1 (M-O, S) probes. Dorsal views with anterior to the left. A’-O’) Transverse views at the level pointed by the black arrowhead of embryos displayed in (A-O). P-S) Transverse views of double in situ hybridized embryos with the indicated probes. ov, otic vesicle; r, rhombomere. (TIF) [file pone.0228225.s001.tif]

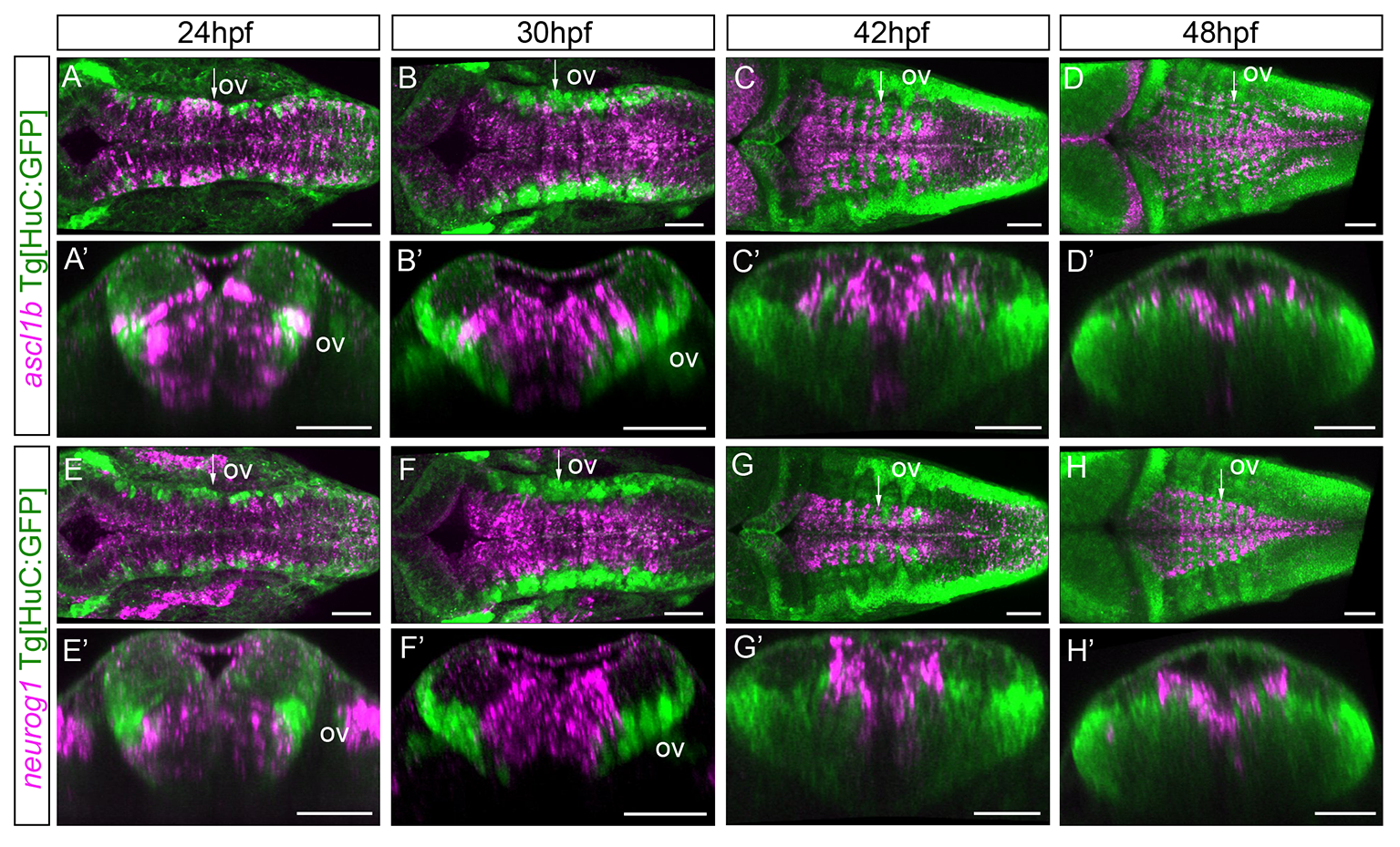

Supplement: S2 Fig — Tg[HuC:GFP] embryos were in situ hybridized with ascl1b (A-D) or neurog1 (E-H) from 24hpf until 48hpf. A-H) Dorsal views with anterior to the left; A’-H’) Reconstructed transverse views at the level pointed by the white arrow in (A-H). Note that progenitor domain in magenta diminishes in size and constitutes the ventricular zone as neuronal differentiation increases over time. ov, otic vesicle. Scale bars correspond to 50 μm. (TIF) [file pone.0228225.s002.tif]

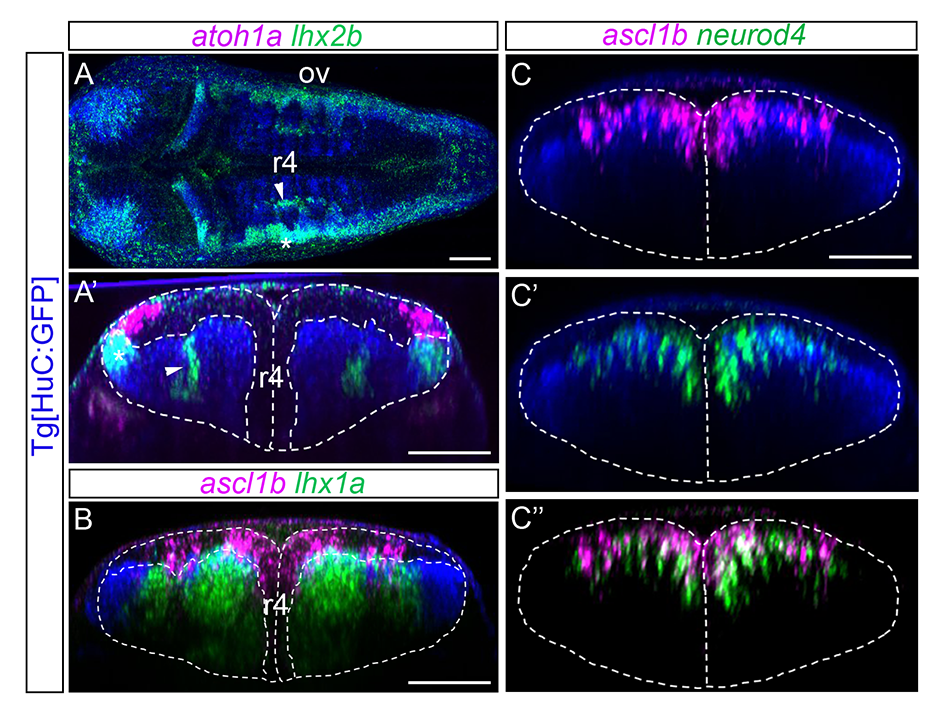

Supplement: S3 Fig — Tg[HuC:GFP] embryos were in situ hybridized either with atoh1a and lhx2b (A-A’), ascl1b and lhx1a (B), or ascl1b and neuroD4 (C-C”). Reconstructed transverse views except for (A), which is a dorsal view, showing the distinct position of progenitors (atoh1a or ascl1b in magenta) and differentiated neurons (lhx2b and lhx1a in green), and cells transitioning towards differentiation (neuroD4 in green) along the DV axis. ov, otic vesicle; r, rhombomere. Scale bars correspond to 50 μm. (TIF) [file pone.0228225.s003.tif]

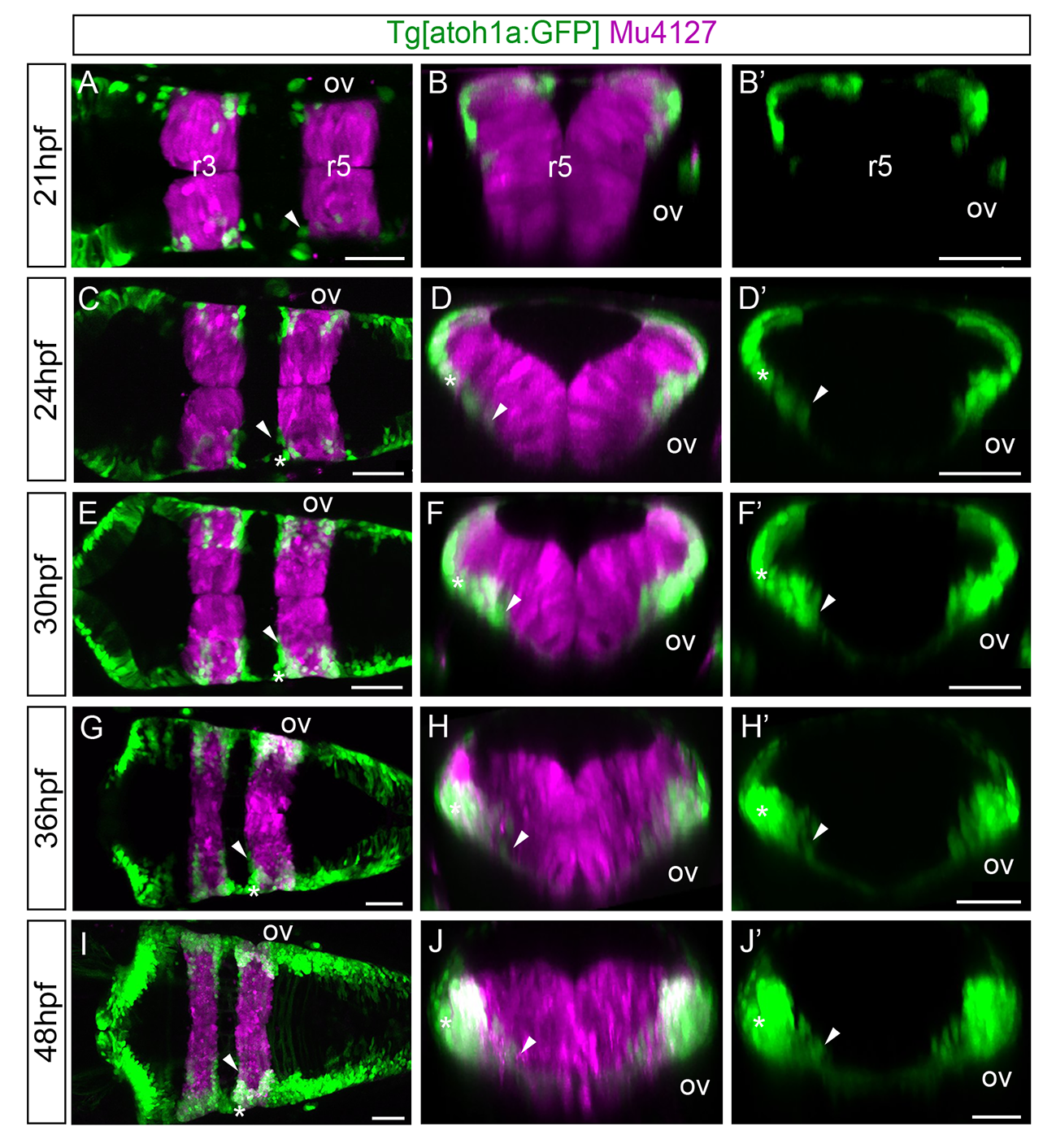

Supplement: S4 Fig — A-E) Double transgenic Tg[atoh1a:GFP]Mu4127 embryos were in vivo imaged at different developmental stages. Dorsal views of confocal MIP from ventral hindbrain with anterior to the left. Note that most of the first born atoh1a:GFP cells (green) at 21hpf position at the rhombomeric boundaries as indicated by the magenta staining in r3 and r5 (see white arrowheads indicating the most ventral atoh1a:GFP derivatives). Later, more atoh1a:GFP cells are generated and populate the whole AP axis (see white asterisks in (B-E)) piling up with the first-born atoh1a:GFP cells (see white asterisks). A’-E’, A”-E”) Reconstructed transverse views of (A-E) at the level of r4/r5 displaying either the two channels (A’-E’) or only the green one (A”-E”). See how the atoh1a:GFP cells corresponding to atoh1a-derivatives end up generating a neuronal arch-like structure (see white arrowheads) as development proceeds. ov, otic vesicle; r, rhombomere. Scale bars correspond to 50 μm. (TIF) [file pone.0228225.s004.tif]

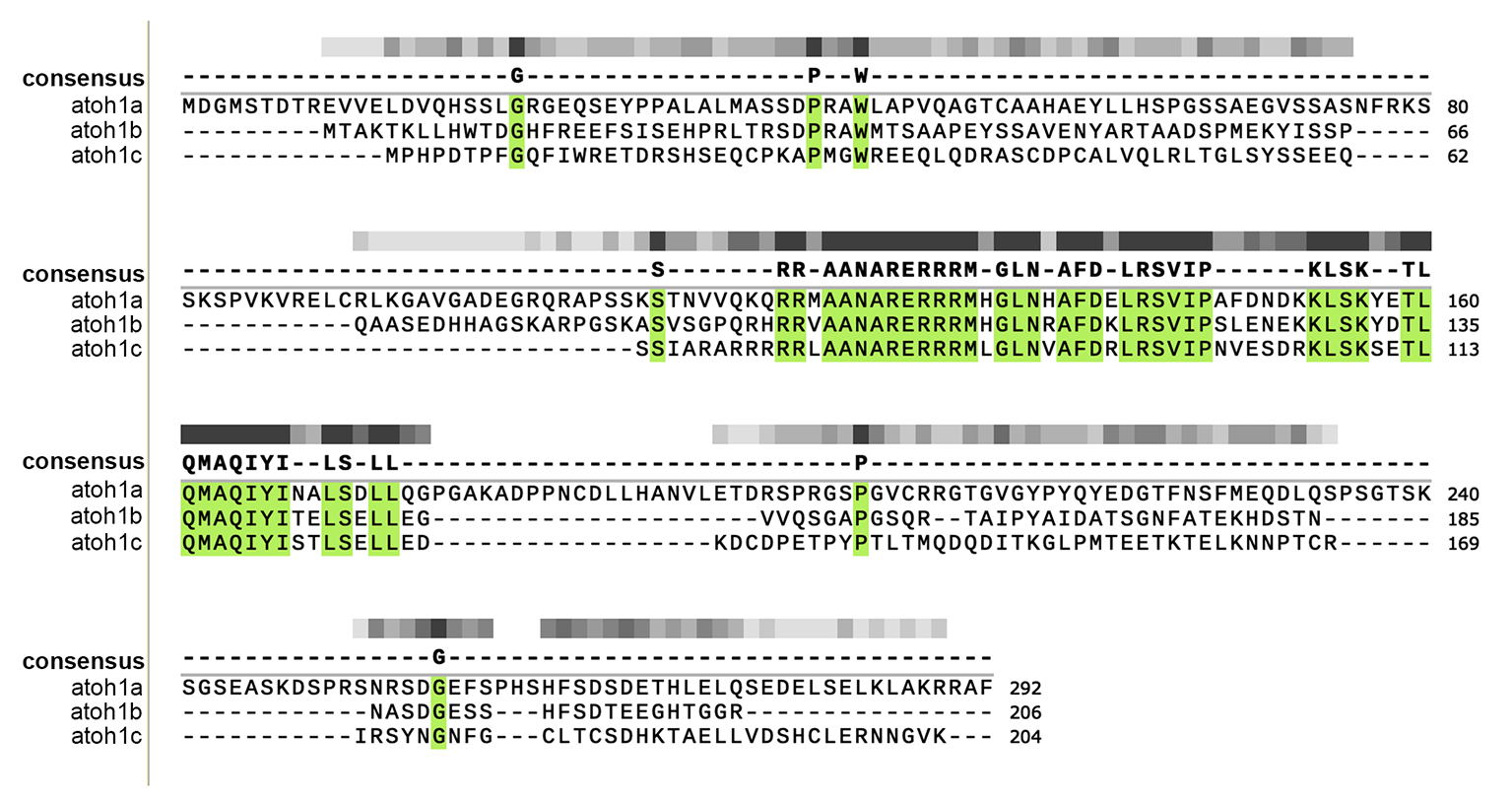

Supplement: S5 Fig — Comparison of zebrafish atoh1a, atoh1b and ato1hc proteins by Multiple Sequence Alignment CLUSTALW (MSA, EMBL-EBI). Sequence conservation (>70%) is displayed at the top as grey blocks with different hues. Amino acids highlighted in green correspond to those that match with the consensus sequence, which is displayed at the top in bold. Note how the three atoh1 proteins are conserved in the central regions and their sequence diverge in the N- and C-terminal domains. (TIF) [file pone.0228225.s005.tif]
