# Supplementary material for: The interplay of atoh1 genes in the lower rhombic lip during hindbrain morphogenesis
Source: PLoS One. 2020 Feb 3;15(2):e0228225. doi: 10.1371/journal.pone.0228225 (PMC6996848; doi:10.1371/journal.pone.0228225)
Supplement: Attachment — Submitted filename: RTR_Belzunce et al.docx [file pone.0228225.s006.docx]

**RESPONSES TO REVIEWER S**

**PONE-D-19-29016: Belzunce et al**

We thank the Reviewers and the Academic Editor for commenting on our paper in order to improve it. We have followed their advices and implemented their suggestions. We improved and modified previously existing figures including new experiments (Figure 1, Figure 5, Figure 7) and incorporated one new figure (Figure S5) to better support our findings. We provide an amended manuscript that I wish it will be suitable for publication in PLOSone.

The responses to the points raised by the Referees are as follows:

**Reviewer 1**

1. We have discussed further our observation that in the absence of atoh1a function progenitors are suspended in a post-mitotic but undifferentiated state and included in the Discussion section (p26).

2. We have investigated whether the effect of premature differentiation of atoh1a-expressing progenitors upon Notch inhibition depended on atoh1a and included these new results in Fig 7I-K. Briefly, we inhibited Notch-signaling in *atoh1a^fh282^*Tg[atoh1a:GFP] mutant sibling embryos, and as predicted neuronal progenitors failed to reach the most ventral territories. We discussed this further after describing the results (p21).

3. We followed the suggestion of the Reviewer of deleting the section in the Discussion about interkinetic nuclear migration and how actin may generate forces for nuclear movement and how that relates to *atoh1b*.

All minor points have been addressed.

**Reviewer 2**

1. The Reviewer suggested to include BrdU-incorporation experiments to demonstrate that indeed *atoh1b* cells were mainly postmitotic.

We followed the Reviewers advice and performed BrdU-incorporation experiments in Tg[atoh1a:GFP] embryos to demonstrate that *atoh1b* cells did not enter S-phase. We included these experiments in Figure 1L-L’’.

2. The Referee wants us to address whether *atoh1b* is required for *atoh1a*-dependent neuronal differentiation of the LRL cells.

We have followed the suggestion of the Referee and performed LOF experiments for atoh1a or atoh1b. For this, we used translation blocking atoh1a and atoh1b morpholinos (Millimaki et al, Development 2007). As provided in the following figure, LOF of atoh1b in the HuC:GFP background results in a similar phenotype to MO-atoh1a injected embryos. In our opinion. These results are in the same line with the ones provided in the manuscript, strongly suggesting that indeed *atoh1b* is downstream of *atoh1a* in the transition of atoh1a-progenitors toward differentiation.

**Fig. Loss-of-function of *atoh1a* and *atoh1b* results in a decrease of differentiated neurons.** Tg[HuC:GFP] embryos were injected with: (A) control-MO (n = 24), and (B) translation-blocking morpholino for atoh1a (n = 21), or (C) for atoh1b (n = 20), and analyzed at 42hpf for LRL-derived neuronal arch-like structures (see white arrow in A). Differentiated neurons in green. D) Graphical representation of the phenotype expressivity upon morpholino-injection. Note that none of embryos injected with the control MO displayed an aberrant phenotype (n = 24/24). For atoh1a-morphants, 85% of the embryos had no LRL-derived neuronal arch-like structures as the embryo displayed in (B) (n = 18/21), and 15% showed partial defects (n = 3/21). atoh1b-MO injected embryos showed a disperse distribution with 40% unaffected (n = 8/20), 40% with a mild phenotype (n = 8/20) and 20% with a strong phenotype similar to atoh1a-morphants as embryo displayed in (C) (n = 4/20). Scale bars correspond to 50μm.

3. The Reviewer raises the interesting aspect of whether loss of *atoh1a* suppresses cell differentiation or cell death.

In our opinion, the comparison of the total number of LRL atoh1a:GFP cells, and the number of them undergoing mitosis in wild type and *atoh1a* mutant siblings (Fig 5A-B) clearly suggested that apoptosis could not be playing a major role in the decrease of the number of neurons. However, we followed the advice of the Referee and now we provide a comparison of the apoptotic events occurring in wild type and *atoh1a^fh282^* mutant embryos. Results show that there is no substantial contribution of apoptosis to the observed mutant phenotype. We described these results in p17 and displayed them in Fig 5C-D and Table 2.

3. The Referee wonders why the atoh1a:GFP expression in the ventricular zone did not increase in the atoh1a mutants.

As it was previously described in Kidwell et al, Dev Biol 2018, the *atoh1a^fh282^*
mutation results in the upregulation of the *atoh1a* mRNA due to the self-regulation of proneural genes transcription. The atoh1a:GFP does not change because it is a genetic tracer of the cells that should be expressing *atoh1a* but they do not, not of the *atoh1a* mRNA.

4. The Reviewer considers that Notch-pathway could regulate either the transition from neural stem cells to neuronal progenitors, or the transition of *atoh1a* progenitors towards differentiation.

The Reviewer is absolutely right and to respond to this question, we conditionally inhibited the Notch-pathway in embryos where *atoh1a* was mutated, and therefore no cells in the LRL could be transitioning towards differentiation. Upon LY-treatment, *atoh1a^fh282^*Tg[atoh1:GFP] embryos displayed a similar phenotype than non-treated mutant embryos, namely: *atoh1a* expression increased, *atoh1b* expression was highly diminished, and GFP-expressing progenitor cells failed to reach the neuronal differentiation domain. These experiments suggested that the transition of *atoh1a* progenitors towards differentiation depends on *atoh1a* function and is regulated by Notch. These new results are described in p21 and included in Fig 7I-K.

Minor comments

1. We rephrased the sentence “We revealed that atoh1a behaves as the cell fate selector gene”, which in Referee’s opinion was too strong (p2, p24) and wrote “We revealed that atoh1a may behave as the cell fate selector gene”.

2. We substituted “atoh1a-derived neurons” by “atoh1a-expressing progenitors” in p13.

3. We categorized cell divisions to PP, NN and NP by position, either in the ventricular zone (P), or in the mantle/differentiation zone (N), and explained it properly in the Fig 3 legend.

4. We examined that atoh1a:GFP cells give rise to HuC-neurons in Fig 2. In the *atoh1a* mutants we did analyze atoh1a:GFP cells by their position in the mantle zone.

5. We have better discussed the difference in the structure of atoh1a and atoh1b proteins in p24 and included a new figure with the amino acid sequence comparison of all atoh1 proteins in zebrafish to support this part of the discussion (Fig S5).
